# Michigan State Veterinary Board

**Published:** 1901-09

**Authors:** 


MICHIGAN STATE VETERINARY BOARD.

The State Veterinary Board of Michigan met in Lansing on August 6 and 7, 1901, for the examination of non-graduates for registration under the veterinary law. There were five applicants who took the examination, of whom two were granted licenses. The Board also passed on the applications of nine graduates and granted them licenses.

